# Simulating Two-Phase Seepage in Undisturbed Soil Based on Lattice Boltzmann Method and X-ray Computed Tomography Images

**DOI:** 10.3390/s24134156

**Published:** 2024-06-26

**Authors:** Zhenliang Jiang, Yiqian Lin, Xian Chen, Shanghui Li, Peichen Cai, Yun Que

**Affiliations:** 1 Department of Civil and Environmental Engineering, The Hong Kong University of Science and Technology, Clear Water Bay, Hong Kong 999077, China; zhenliang.jiang@connect.ust.hk; 2 College of Civil Engineering, Fuzhou University, Fuzhou 350116, China; c450639865@163.com (X.C.); peichencai@chd.edu.cn (P.C.); queyun_2001@fzu.edu.cn (Y.Q.); 3 College of Intelligent Construction, Fuzhou University of International Studies and Trade, Fuzhou 350202, China; lsh@fzfu.edu.cn

**Keywords:** lattice Boltzmann method, X-ray computed tomography, undisturbed soil, two-phase flow, wettability of pore walls

## Abstract

The two-phase seepage fluid (i.e., air and water) behaviors in undisturbed granite residual soil (U-GRS) have not been comprehensively studied due to a lack of accurate and representative models of its internal pore structure. By leveraging X-ray computed tomography (CT) along with the lattice Boltzmann method (LBM) enhanced by the Shan–Chen model, this study simulates the impact of internal pore characteristics of U-GRS on the water–gas two-phase seepage flow behaviors. Our findings reveal that the fluid demonstrates a preference for larger and straighter channels for seepage, and as seepage progresses, the volume fraction of the water/gas phases exhibits an initial increase/decrease trend, eventually stabilizing. The results show the dependence of two-phase seepage velocity on porosity, while the local seepage velocity is influenced by the distribution and complexity of the pore structure. This emphasizes the need to consider pore distribution and connectivity when studying two-phase flow in undisturbed soil. It is observed that the residual gas phase persists within the pore space, primarily localized at the pore margins and dead spaces. Furthermore, the study identifies that hydrophobic walls repel adjacent fluids, thereby accelerating fluid movement, whereas hydrophilic walls attract fluids, inducing a viscous effect that decelerates fluid flow. Consequently, the two-phase flow rate is found to increase with then-enhanced hydrophobicity. The apex of the water-phase volume fraction is observed under hydrophobic wall conditions, reaching up to 96.40%, with the residual gas-phase constituting 3.60%. The hydrophilic wall retains more residual gas-phase volume fraction than the neutral wall, followed by the hydrophobic wall. Conclusively, the investigations using X-ray CT and LBM demonstrate that the pore structure characteristics and the wettability of the pore walls significantly influence the two-phase seepage process.

## 1. Introduction

Soil is a natural construction material that consists of three phases: soil particles, water, and air [1,2,3]. The infiltration of water into soil results in a process known as two-phase flow, which has been extensively studied in various engineering fields such as oil extraction, waste treatment, and groundwater pollution [4,5]. Experimental techniques using different sensors, such as two-dimensional (2D, e.g., [6,7,8]) and three-dimensional (3D, e.g., [9,10,11,12,13]) methods, have been used to examine two-phase flow. However, these methods are destructive, costly, and not easily repeatable. To address these limitations, numerical simulation methods at the pore scale have been developed because they offer the advantages of repetitiveness, convenience, and visualization when simulating the dynamic displacement process within the pore structure [14,15,16,17]. Currently, the numerical simulation methods used for multiphase flow at the pore scale include level set, phase field, lattice Boltzmann method (LBM), and others [18,19].

Although the level set and phase field methods have been used in studies, most of them fall under finite element simulation, which assumes that the research object is a continuous medium. In reality, the solid and water phases are discrete entities, and the continuous medium approach inevitably deviates from the actual pore structure. The LBM is a mesoscopic method that is discrete at the macroscopic level and continuous at the microscopic level. It discretizes the computational region into a number of lattices, with the lattice scales between the molecular mean free travel and the control cell in the finite volume method, thus abstracting the fluid flow into a large number of microscopic particles colliding and transporting according to the established rules [20]. The lattice Boltzmann method was first proposed by McNamara et al. [21] and is now widely used in the field of numerical simulation of two-phase flow, such as the color function model [22], free energy model [23], and Shan–Chen pseudo-potential model [24,25,26,27]. The Shan–Chen model, renowned for its facile coupling with microscopic interaction forces, accurately embodies the intrinsic physical characteristics of two-phase fluid dynamics. Its straightforward programmability further contributes to its extensive application in the field. Numerous scholars have utilized the Shan–Chen model, as demonstrated in a series of studies [28,29,30,31,32,33,34], to simulate the two-phase immiscible repulsion process on the pore scale. For instance, Shan et al. [28] investigated the optimal displacement of immiscible two-phase fluids in a pore doublet using the Shan–Chen model. Hosseini et al. [29] employed the multiphase LBM to investigate the source of hysteresis in the soil–water characteristic curve. Liu et al. [30] studied local instabilities during capillary-dominated immiscible displacement in porous media. Wang et al. [31] and Wang et al. [32] conducted multiphase LBM to analyze cyclic water retention behavior and porosity variation on water retention behavior, respectively. Additionally, Takken and Wille [33] conducted a simulation study on pressure-driven and channel-based microfluidics. Hu [34] studied the soil water distribution from the perspective of the pore-scale using LBM. These references provide valuable insights into the application of the Shan–Chen model and the multiphase LBM in understanding the two-phase immiscible repulsion process at the pore scale. However, the majority of these studies have centered around the investigation of the multiphase flow of rock and soils. Scant attention has been given to the study of undisturbed soil.

The intricate internal pore architecture of natural soils, exemplified by granite residual soil (GRS) prevalent in the southeast coastal region of China, is subject to a multitude of influences [35]. These soils experience significant alterations owing to factors such as the intrusion of vegetation roots, the formation of animal burrows, and the cyclic impact of moisture variations, among others, causing the generation of macro-pores and sensitivity to the disturbance [35,36]. Therefore, it is difficult to accurately describe the pore characteristics of the interior of the GRS using traditional destructive experimental tests.

Currently, the integration of non-destructive X-ray CT scanning and image processing technologies facilitates the extraction of quantitative attributes of soil pore structure without destroying the original soil structure and is regarded as one of the most effective non-destructive methods [37,38,39,40]. This advancement plays a pivotal role in constructing accurate pore models of authentic soil samples, offering significant insights into the physical characteristics of the soil pore structure [41]. Therefore, carrying out numerical simulation of multiphase flow on the basis of the X-ray CT images helps to characterize the pore fluid transport, which is of great significance to further reveal the mechanism of water–gas two-phase seepage in the soil pore.

Hence, this paper aims to use reconstructed CT images of a undisturbed GRS (U-GRS) and the Shan–Chen model in the lattice Boltzmann method to simulate the two-phase seepage replacement process by MATLAB programming. The study aims to investigate the dynamic visualization of the two-phase flow process within the pore space and analyze the influence of the factors of the pore structure, porosity, and wall wettability on the replacement process. The paper provides a comprehensive understanding of the two-phase flow process in the U-GRS, which can be used to develop effective strategies for soil management and engineering applications.

## 2. Undisturbed Soil Sampling and CT Scanning

**Figure 1:**
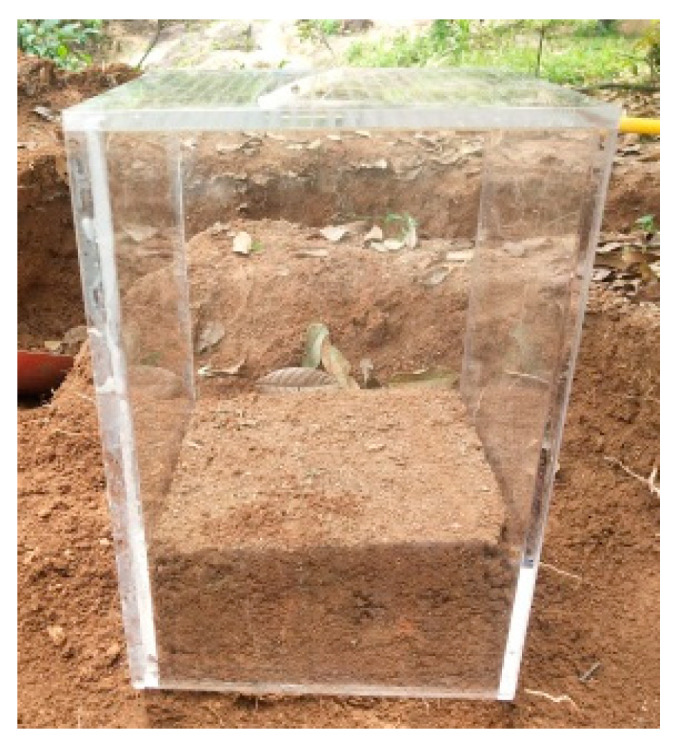
Sampling of the U-GRS [36].

**Figure 2:**
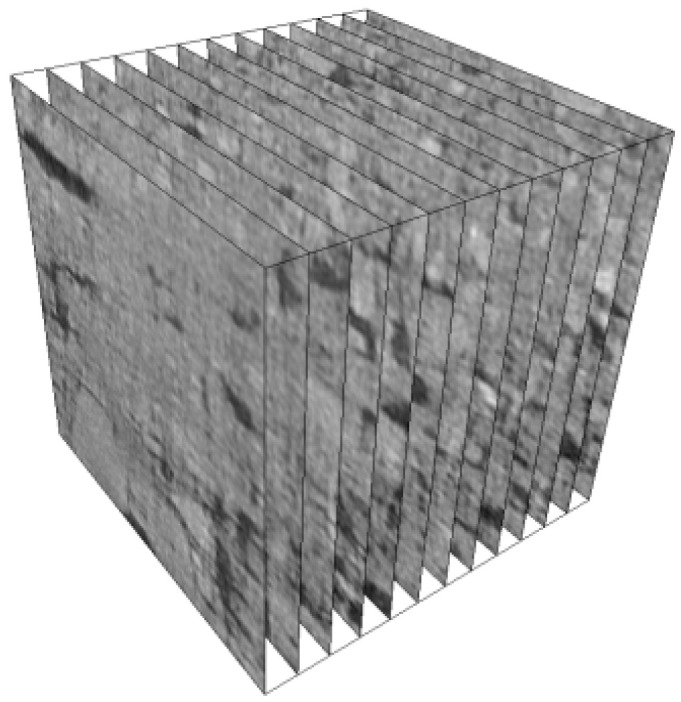
Representative slices of X-ray CT images of a U-GRS sample.

The test site was selected from a densely vegetated GRS slope in the northwest of Fuzhou, China. The surface layer of the slope in the test area contains weeds and the slope is gentle, as shown in Figure 1, and a more detail description can be found in the literature [35]. The schematic diagram of the undisturbed soil sampling process. The CT slices of U-GRS samples were collected with a C450KV high-energy industrial CT scanner of Shanghai Yinghua Testing Company. Representative slices of X-ray CT images of the soil samples are illustrated in Figure 2. The scanning parameters were set as follows: the working voltage was 450 kV, the current was 63 mA, and the scanning resolution was 0.15 mm [35].

## 3. Basic Theory of the Two-Phase Lattice Boltzmann Model

In this paper, the two-phase seepage simulation of U-GRS adopts the D2Q9 model of lattice Boltzmann model. The collision operator is BGK approximation, and the evolution equation is Formula (1), as follows:

(1) $$F_{\alpha }(\omega +e_{\alpha }\delta_{t},t+\delta_{t})=F_{\alpha }(\omega ,t)-\frac{F_{\alpha }(\omega ,t)-F_{\alpha }^{\mathrm{eq}}(\omega ,t)}{\tau }$$

where $F_{\alpha }(\omega ,t)$ is the particle distribution function along the α-direction at the lattice point *ω* at time t; ***e****_α_* is the discrete velocity; *δ_t_* is discrete-time; *τ* is the dimensionless relaxation time; $F_{\alpha }^{\mathrm{eq}}(\omega ,t)$ is the local equilibrium state distribution function in discrete velocity space.

### 3.1. Potential Model

The two-phase lattice Boltzmann model, enhanced by the Shan–Chen model, is introduced in the following as an example of the D2Q9 model [38]. The model assumes that there are *S* components, and the form of the LBM equation for the *k*th component is as follows:

(2) $$f_{i}^{k}(x+e_{i},t+1)=f_{i}^{k}(x,t)+\Omega_{i}^{k}(x,t),$$

To simplify the collision term, a linearized single relaxation time form is used:

(3) $$\Omega_{i}^{k}(x,t)=-\frac{1}{\tau_{k}}(f_{i}^{k}(x,t)-f_{i}^{k(\mathrm{eq})}(x,t))$$

where $f_{i}^{k}(x,t)$ is the distribution function of component *k* at point *x* at time *t*; *k* = 1~*S* denotes the component; *i* = 0, 1, 2, …, 8 denotes the direction; *τ_k_* denotes the average collision time of phase *k* and determines the viscosity of the fluid in phase *k*; and $f_{i}^{k(\mathrm{eq})}(x,t)$ is the corresponding equilibrium distribution function of the following form:

(4) $$f_{i}^{k(\mathrm{eq})}=\left\{\begin{array}{l} \alpha_{k}n_{k}-\frac{2}{3}n_{k}(u_{k}^{eq}{)}^{2},i=0\\ \frac{(1-\alpha_{k})n_{k}}{5}+\frac{1}{3}n_{k}(e_{i}u_{k}^{eq})+\frac{1}{2}n_{k}(e_{i}u_{k}^{eq}{)}^{2}-\frac{1}{6}n_{k}(u_{k}^{eq}{)}^{2},i=1,2,3,4\\ \frac{(1-\alpha_{k})n_{k}}{20}+\frac{1}{12}n_{k}(e_{i}u_{k}^{eq})+\frac{1}{8}n_{k}(e_{i}u_{k}^{eq}{)}^{2}-\frac{1}{24}n_{k}(u_{k}^{eq}{)}^{2},i=5,6,7,8 \end{array}\right.,$$

where *e_i_* is the discrete velocity vector; *α_k_* is the free parameter, seen as $c_{s}^{k}=0.6\times (1-\alpha_{k})$ in reference [41], where $(c_{s}^{k}{)}^{2}m_{k}=1/3$, *m_k_* ≥ 1.

The intermolecular forces change their distribution by affecting the equilibrium state velocity as described in the following equation:

(5) $$\rho^{k}u^{k(\mathrm{eq})}=\rho^{k}u^{\prime }+\tau_{k}F_{k},$$

where $\rho_{k}=m_{k}n_{k}$, and *m_k_* is the molecular mass of *k* phase.

To satisfy conservation of momentum during the collision and balance the *u*′ term in the velocity, it is redefined as follows:

(6) $$u^{\prime }=\frac{\sum_{k=1}^{s}\frac{\rho^{k}u^{k}}{\tau_{k}}}{\sum_{k=1}^{s}\frac{\rho^{k}}{\tau_{k}}},$$

*F_k_* consists of three parts: *F*_1*k*_ is the interaction force between the *k*th component and the other components; *F*_2*k*_ is the interaction force between the *k*th component and the wall; and *F*_3*k*_ is the mass force on the *k*th component [42]. The calculation equation is as follows:

(7) $${F_{1}}_{k}=-\psi_{k}(x)\sum_{x^{\prime }}\sum_{k=1}^{s}G_{k\bar{k}}(x,x^{\prime })\psi_{\bar{k}}(x^{\prime })(x^{\prime }-x),$$

(8) $$G_{k\bar{k}}(x,x^{\prime })=\left\{\begin{array}{l} g_{k\bar{k}},\;\left|x^{\prime }-x\right|=e_{i}(i=1,2,3,4,)\\ \frac{g_{k\bar{k}}}{4},\;\left|x^{\prime }-x\right|=e_{i}(i=5,6,7,8)\\ 0,\;others \end{array}\right.,$$

where $G_{k\bar{k}}$ is the interaction strength of neighboring particles between components; $\psi_{k}(x)$ is a function of *n_k_*(*x*), which is generally taken directly as *n_k_*(*x*).

(9) $${F_{2}}_{k}=-\psi_{k}(x)\sum_{x^{\prime }}g_{kw}n_{w}(x^{\prime })(x^{\prime }-x),$$

where *n_w_* is the wall quantity density.

*g_kw_* is the force parameter between the *k*th identical wall surfaces: when *g_kw_* > 0, the *k*th phase is non-wetted and when *g_kw_* < 0, it is wetted [42].

(10) $$F_{3k}=m_{k}n_{k}g,$$

where *g* is the force per unit mass.

The continuity and momentum equations of the mixed fluid can be obtained by expanding them using the Chapman–Enskog method [43]:

(11) $$\frac{\partial \rho }{\partial t}+\nabla (\rho u)=0,$$

(12) $$\rho [\frac{\partial u}{\partial t}+(u\nabla )u]=-\nabla p+\nabla [\rho \mu (\Delta u+u\Delta )]+\rho g,$$

where mixing density *ρ*, velocity *u*, pressure *p*, and dynamic viscosity *μ* are calculated as shown in the following equations:

(13) $$\rho =\sum_{k}\rho_{k},$$

(14) $$\rho u=\sum_{k}\rho_{k}u_{k}+0.5\sum_{k}F_{k},$$

(15) $$p=\frac{1}{3}\sum_{k}n_{k}+\frac{3}{2}\sum_{k,\bar{k}}G_{k\bar{k}}\psi_{k}\psi_{\bar{k}},$$

(16) $$\mu =\frac{1}{3}(\sum_{k}\frac{\rho_{k}}{\rho }\tau_{k}-\frac{1}{2}),$$

### 3.2. Boundaries

**Figure 3:**
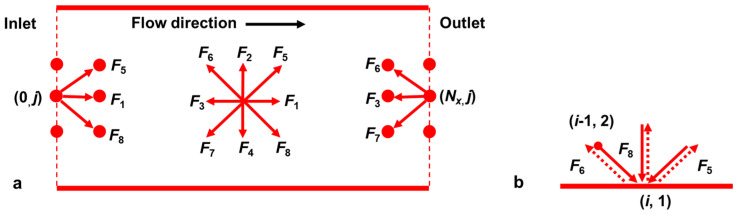
Boundaries: (**a**) periodic boundary; (**b**) bounce scheme.

As illustrated in Figure 3, the Shan–Chen pseudopotential model is used for the simulation of two-phase seepage flow field, with periodic boundary for the inlet and outlet boundary conditions, and standard rebound-format boundary for the inter-particle migration process inside the pore, which will be elaborated in the following.

The periodic boundary defines that the model pore is infinitely long in the seepage direction, and the fluid particles enter the flow field from the inlet boundary, leave the flow field from the outlet boundary, and continue to enter the flow field from the inlet boundary in the next time step when they leave the flow field [44]. The specific implementation of the process is carried out as follows. As can be clearly seen in Figure 3a, the particle distribution function for the inlet boundary can be obtained from the migration of the outlet boundary, i.e.,:

(17) $$\left\{\begin{array}{c} F_{1}(0,j,t+\Delta t)=F_{1}(N_{x},j,t)\\ F_{5}(0,j,t+\Delta t)=F_{5}(N_{x},j,t)\\ F_{8}(0,j,t+\Delta t)=F_{8}(N_{x},j,t) \end{array}\right.,$$

Similarly, the particle distribution function at the exit boundary can be obtained as follows:

(18) $$\left\{\begin{array}{c} F_{3}(N_{x},j,t+\Delta t)=F_{3}(0,j,t)\\ F_{6}(N_{x},j,t+\Delta t)=F_{6}(0,j,t)\\ F_{7}(N_{x},j,t+\Delta t)=F_{7}(0,j,t) \end{array}\right.,$$

The standard bounce is used to simulate the flow behavior without slipping between solid particles of soil and fluid during seepage [44]. The fluid node is incident to (*α*, *t*) from (*α*−1, *t*) to the distribution function *F*_α1_, the migrating collision returns in the original way, which obtains the *F_α_*_0_ on the node (*α*, *t*), and the other nodes are similar, presented in Figure 3b. That is, the distribution function can be expressed as follows:

(19) $$F_{\alpha 0}(x_{b},t)={F_{\alpha }}_{1}(x_{f},t)$$

where *α*_1_ is the direction toward the wall; *α*_0_ is the opposite direction of *α*_1_; *x_b_* is the solid wall lattice point; *x_f_* is the fluid lattice point, and *x_f_* = *x_b_ − e_α_δ_t_*.

### 3.3. Computational Procedures

**Figure 4:**
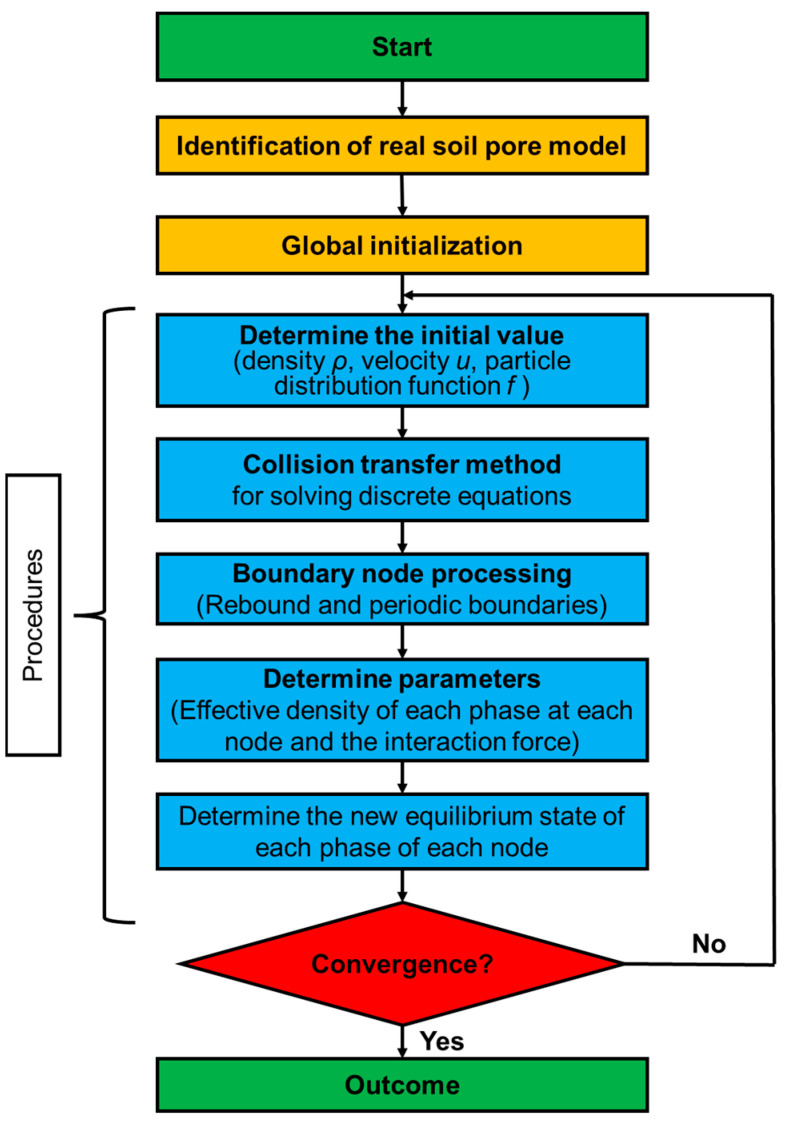
Calculation flowchart of the two-phase seepage flow based on LBM by incorporating Shan–Chen model.

The Shan–Chen pseudo-potential model has been favored by more scholars by virtue of its ease in revealing the nature of the dynamics between multiphase multicomponent at microscopic scale [41]. The computational flow is shown in Figure 4.

## 4. Verification of Two-Phase Lattice Boltzmann Model Program

This section provides a detailed description of the two-phase flow model validation process, which consists of two main parts: the two-phase separation validation model and the single-orifice two-phase repulsion validation model.

### 4.1. Validation of Two-Phase Separation Model

A 200 × 200 grid area is selected as the two-phase seepage validation computational model, all parameters in the model are in lattice units, and the surrounding boundary conditions are processed with periodic boundary conditions to simulate the two-phase separation process under the three kinds of force strengths *G*. The specific computational parameters are shown in Table 1.

**Figure 5:**
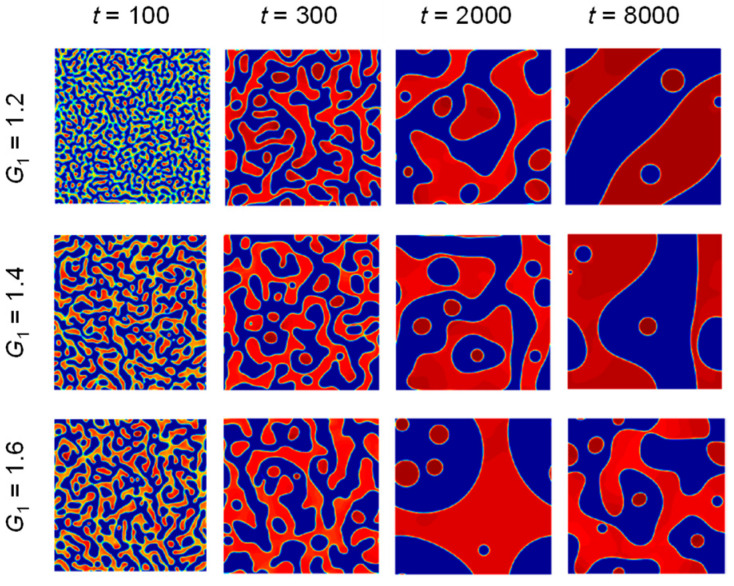
Schematic diagram of *G*_1_ = 1.2, 1.4, and 1.6 two-phase separations (unit: step).

The two-phase separation process was simulated for the three cases of *G*_1_ = 1.2, *G*_2_ = 1.4, and *G*_3_ = 1.6, respectively, and the changes in the density distribution during the calculation process are shown in Figure 5. It can be found that with the increase in time, the two intermixed fluids are gradually separated, and with the increase in the strength of the interaction with the components *G* from 1.2 to 1.6, the speed of phase separation and polymerization increases, the phase interface is also gradually narrowed, and the transition region becomes clearer; the above analysis shows that the size of the value of *G* has an important influence on the degree of intermiscibility between the components.

### 4.2. Validation of a Two-Phase Repulsion Model for a Single Orifice

The Shan–Chen model is used to validate the two-phase immiscible displacement process in a single pore channel [45]. A grid area of 330 × 60 is selected as the computational model for the validation of two-phase seepage in a single pore channel, and all parameters in the model are in lattice units, with the same boundary conditions as in Section 3.2. At the initial moment of simulation, the channel is filled with phase 1 (blue phase) with a density of 0.0001, and the simulation starts with phase 2 (red phase) driving from the inlet with a density *ρ* of 1.0, a kinematic viscosity coefficient *μ* of 1.0, and a *G* of 0.12; the interaction parameters between the fluid and the solid wall are *G_w_*_1_ = −0.02 and *G_w_*_2_ = 0.02, and the inlet velocity *u* = 0.2.

**Figure 6:**
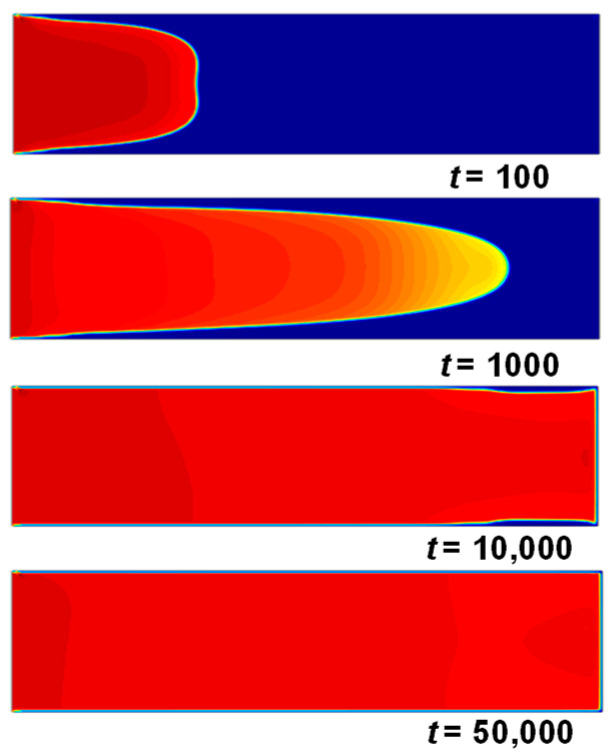
Two-phase immiscible displacement process in a single channel (unit: step).

Schematic diagrams of the two-phase expulsion process at 100, 1000, 10,000, and 50,000 time steps are given in Figure 6. As can be found, the interface between the two phases gradually changes from flat to rounded, the degree of curvature increases, and the phenomenon of “fingering” is particularly significant. At the late stage of the replacement *t* = 10,000 time step, it can be observed that there are still a few replaced phases near the upper and lower walls of the replacement phase. The reason is that the existence of pseudo-velocity at the interface of the two phases makes the pseudo-potential model calculation appear to be a “false” phenomenon [46], but as shown in *t* = 50,000, with the prolongation of the replacement time, the displaced phase will eventually be displaced out of the channel. The results are consistent with those of the literature [22,32], which verifies the correctness of the replacement model adopted in this paper.

## 5. Results and Discussion

Based on the X-ray CT images of the U-GRS, the seepage of two immiscible phases (water phase and gas phase) in a soil pore space are simulated with the LBM aided by the Shan–Chen pseudo-potential model, and the effects of different factors on the seepage process of the two phases are analyzed.

**Figure 7:**
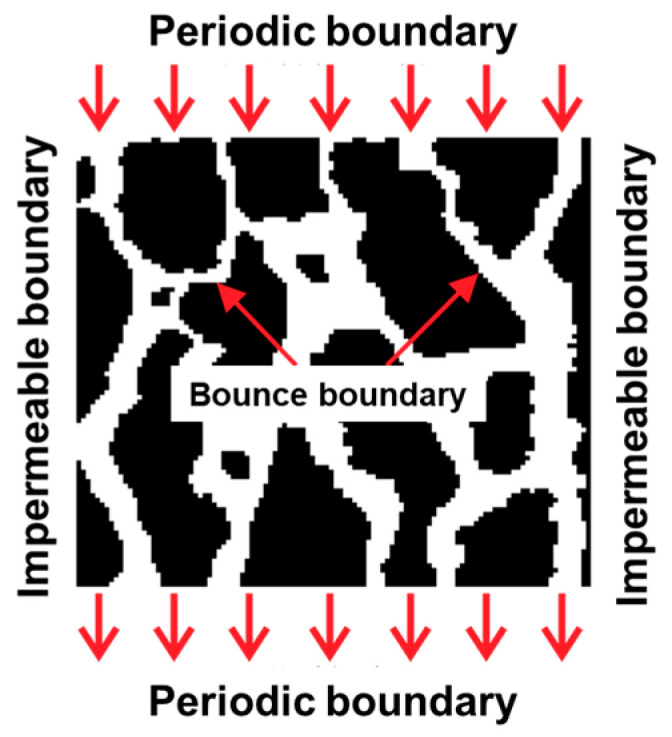
Schematic diagram of the two-phase flow and boundaries. The red arrows mean the flow direction.

The seepage direction in the soil body is set to be along the depth direction of the soil body. The initial moment of the model is filled with the driven phase (gas phase) and the immiscible driven phase (water phase) is injected from the inlet, and the specific boundary conditions of the model are set up as shown in Figure 7. The units and related parameters of two-phase percolation are set according to the literature [22,32], i.e., all the units in this paper are dimensionless lattice units, while the density of the gas phase *ρ*_1_, the density of the water phase *ρ*_2_, the force at the interface between the two phases *G_w_*, the force between the repellent phase and the solid wall *G_wr_*, the force between the driven phase and the solid wall *G_wb_*, the inlet velocity *u*, the lattice step size *δ_x_*, and the time step *δ_t_* are detailed in the parameters shown in Table 2.

### 5.1. Influence of Pore Structure

In order to investigate the influence of the pore structure on the two-phase seepage process, with reference to the binarization method in the literature [3], the pore structures with better connectivity are selected for simulation, as shown in Figure 7. As noted, the porosity *n* is 39.03%, the model size is 100 × 110 pixels, and the mesh computation area is 100 × 110, respectively. The lattice units are used for all the parameters, the boundary conditions are the same as above, and the hydrophobic wall is adopted [31,32] with *G_wr_* = 0.2, *G_wb_* = −0.2 as an example. The other calculation parameters are shown in Table 2.

**Figure 8:**
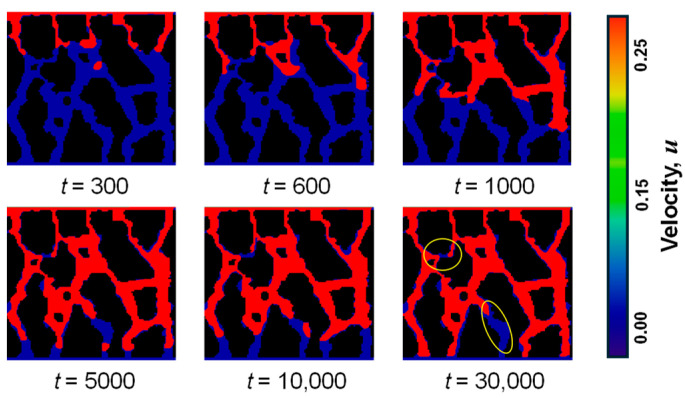
Schematic diagram of two-phase seepage process (hydrophobic wall) (unit: step). The colors indicate the driven phase (gas, shown in blue) and the driving phase (water, depicted in red).

Figure 8 presents a dynamic visualization of two-phase seepage across various temporal intervals, delineating the driven phase (gas phase) in blue and the driving phase (water phase) in red. The forefront of the water-phase seepage demonstrates a “finger-in” progression, manifesting a preference for larger, more direct channels for infiltration, subsequently resorting to narrower, more tortuous pathways. A disruption in flow within certain constricted regions is highlighted by a yellow circle within the figure. This phenomenon can be attributed to two primary factors: firstly, the narrowness of the pore encircled in yellow, which impedes the ingress of water fluid; secondly, the existence of adjacent channels that are comparatively wider and straighter, where the water fluid preferentially migrates, engendering a pronounced pore preferential flow effect. This preferential flow effect has been observed in previous studies, where water preferentially flows through larger and more connected channels, while gas is displaced and trapped in smaller pores [9,47].

Concurrently, as the water phase predominates within the major channels, it also obstructs the expulsion of the gas phase at the interruption points. The presence of water in the channels creates a barrier for the gas phase, preventing its displacement and causing it to be trapped in the smaller pores. This finding is consistent with the observation of sporadic appearances of free water fluids within the gas-phase domain during the two-phase flow process [48]. The discontinuity of the water phase suggests that it does not maintain continuous integrity throughout the displacement of the gas phase. This behavior can be attributed to the detachment of the leading water phase flow from the main water body and its intrusion into the principal gas-phase region in regions characterized by larger pores [32]. Moreover, the gas-water phase flow persists in migrating within the pores until it either merges with the main water body or exits the soil matrix, exerting a relatively minimal displacement impact on the gas phase [32]. This behavior indicates that the displacement of the gas phase by the water phase is not uniform and can be influenced by the pore structure and the preferential flow paths. These findings highlight the complex dynamics of two-phase flow in undisturbed soil and the need to consider the interplay between pore geometry and fluid behavior for a comprehensive understanding of the displacement process.

**Figure 9:**
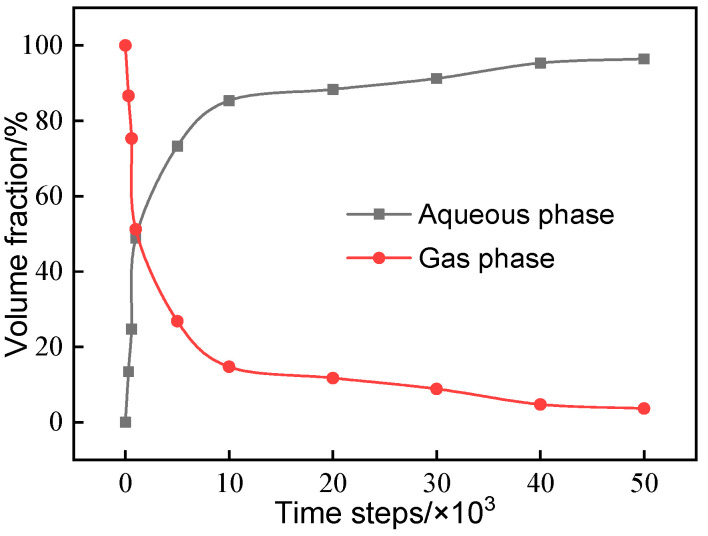
Change of the two-phase volume fractions at different time steps (unit: step).

Figure 9 illustrates the temporal evolution of the volume fraction for a two-phase seepage. Initially, there is a marked increase in the volume fraction of the water phase, juxtaposed with a significant decrease in the gaseous phase volume fraction. This pronounced change in volume fractions is predominantly observed during the early stages of biphasic percolation. Moreover, the rate of change in volume fractions for both phases exhibits a declining trend as the time step progresses. This observation is in concordance with the schematic representation of the two-phase seepage process delineated in Figure 9. Post completion of the two-phase percolation event, residual gas phase presence within the pore spaces is noted. This occurrence can be attributed to the intricate pore structure inherent within the native soil matrix, which impedes the timely infiltration of the displacing water phase into certain constricted pore channels that are interconnected with larger voids, as depicted by the yellow circles in the figure.

### 5.2. Influence of Porosity

**Figure 10:**
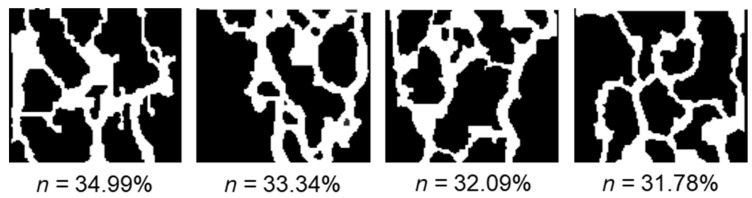
CT images of the GRS with different porosities.

To study the properties of two-phase seepage of water–air at varying porosities, four GRS models with good connectivity and different porosities were selected, as illustrated in Figure 10. The porosities of the models were 34.99%, 33.34%, 32.09%, and 31.78%. These models had a size of 100 × 110 pixels, with a mesh computation area of 100 × 110 lattice points. All the parameters in the model were in lattice units, and the boundary conditions were treated as shown in Figure 7. The two-phase seepage process is also simulated with the hydrophobic wall [31,32] (*G_wr_* = 0.2, *G_wb_* = −0.2), respectively. The other computational parameters are the same as those in Table 2.

**Figure 11:**
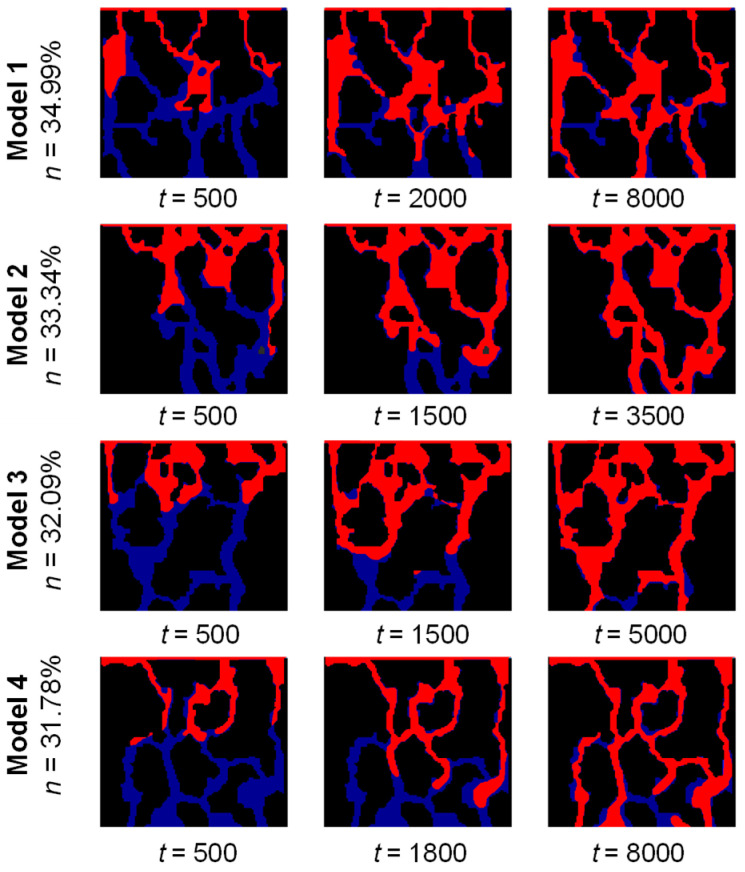
Schematic diagrams of the two-phase seepage process of the GRS models with different porosities (unit: step). The colors indicate the driven phase (gas, shown in blue) and the driving phase (water, depicted in red).

Figure 11 illustrates a step-by-step diagram illustrating the two-phase flow process for various porosity models. The graph indicates that the length of the two-phase flow path and the time needed to complete the process vary for different models with varying porosities. For instance, Model 1 and Model 4 require 8000 time steps, whereas Model 2 only needs 3500 time steps. This is due to the complex pore structures of Model 1 and Model 4, which contain interlaced pores, while Model 2 has relatively straight and single pores with fewer interlaced ones. The morphology of pore structure significantly influences the efficiency of water phase displacement of gas phase during two-phase flow [9,47].

**Figure 12:**
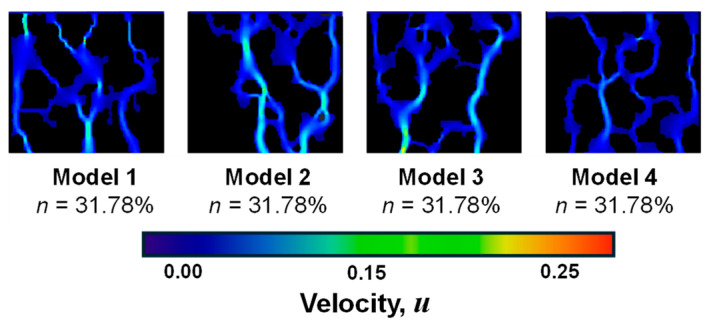
Water-phase seepage velocity field of the GRS models with different porosities while seepage stabilization.

Figure 12 shows the distribution of the water-phase seepage velocity field of different porosity models after the stabilization of two-phase seepage. It can be found that the overall water-phase velocity values show a decreasing trend in turn, with model 1 exhibiting an overall velocity of *u*_1_ = 0.174, model 2 at *u*_2_ = 0.152, model 3 at *u*_3_ = 0.116, and Model 4 at *u*_4_ = 0.062. It indicates that when the seepage is stable, the overall velocity of the water phase decreases as the porosity of the model decreases, and the maximum value of the two-phase seepage velocity is smaller. Besides, Model 2 and Model 3 present a significant large pore preferential flow phenomenon, which is not apparent in the rest models. It indicates that the local seepage velocity is closely related to the pore structure morphology, and the generation of the preferential flow is not significantly correlated to the porosity [9,47,48]. The lack of a significant correlation between preferential flow and porosity highlights the importance of considering the spatial distribution and connectivity of pores when studying two-phase flow in undisturbed soil. Pore structure characteristics, such as the presence of narrow or wide channels, can have a significant impact on the flow behavior and the preferential migration of fluids. Therefore, a comprehensive characterization of the pore structure morphology is crucial for understanding and predicting the flow dynamics in undisturbed soil. These findings have important implications for soil management and engineering applications. Understanding the preferential flow behavior and its relationship with pore structure can help in designing more efficient drainage systems, optimizing irrigation practices, and predicting contaminant transport in soil. By considering the specific pore structure characteristics, engineers and researchers can develop more accurate models and simulations to predict and mitigate potential flow-related issues in soil systems.

### 5.3. Influence of Wall Wettability

After the pore wall contact with water, different physicochemical changes will occur, and due to the immiscibility of different phases, the existence of interfacial tension, resulting in multiphase flow, presents a complex flow pattern [30]. These flow patterns are closely related to the inherent characteristics of the medium and play a decisive role in the multiphase seepage process and seepage characteristics. Hence, the wettability of soil pore wall is an important factor affecting multiphase seepage patterns.

**Figure 13:**
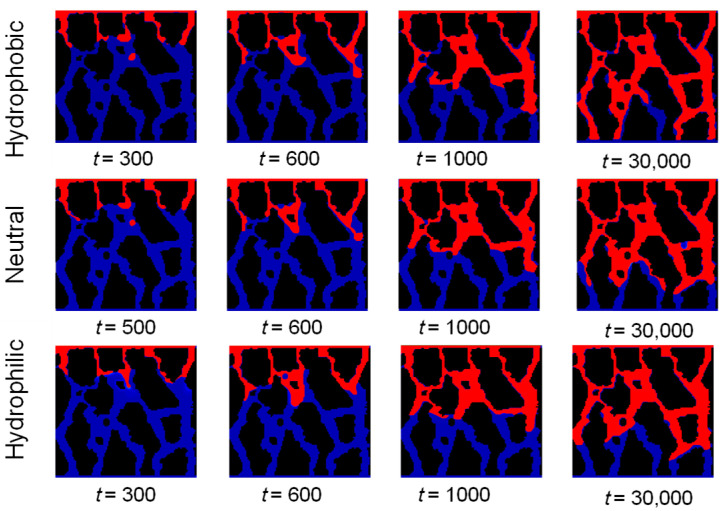
Schematic diagrams of the two-phase seepage process of the GRS model with different wall wettability. The colors indicate the driven phase (gas, shown in blue) and the driving phase (water, depicted in red).

In order to investigate the influence of pore wall wettability on the two-phase seepage process of water and gas, the pore surface of the hydrophobic wall (*G_wr_* = 0.2, *G_wb_* = −0.2), neutral wall (*G_wr_* = 0, *G_wb_* = 0), and hydrophilic wall (*G_wr_* = −0.2, *G_wb_* = 0.2) are used as examples based on the model shown in Figure 7. The settings of the LBM model are the same as those in Section 5.1, where the hydrophobic wall was simulated. This section mainly simulates the two-phase seepage process under the conditions of a neutral wall and a hydrophilic wall. In order to facilitate the comparison of the three conditions, the time step selection is the same as those in Section 5.1, and the results of the calculations are shown in Figure 13.

It can be seen from Figure 13 that it is evident that the replacement phase (water) occupies an increasing pore space throughout the process, while the replacement phase (gas) decreases. However, there is always a residual gas phase in the pore space, mainly located at the pore edge and dead center of the model. The leading edge of the interface is primarily convex arc-shaped, except for a concave arc at the early stage of seepage, particularly when the hydrophilic wall drives the concave leading edge stronger. The reason is that, at the start of seepage, the gas phase’s volume fraction is larger, resulting in greater force on the water phase and increased replacement resistance. Consequently, the front edge of the replacement exhibits a concave shape. This higher gas volume fraction creates a stronger resistance to the displacement of the water phase, leading to a concave shape at the front edge of the replacement. This concave shape indicates that the water phase is being pushed back by the gas phase, resulting in a slower and more uneven displacement process. As the replacement process continues, the front edge becomes stabilized by the pore structure, and the gas phase’s volume fraction decreases significantly. This reduction in gas volume fraction leads to a decrease in replacement resistance, allowing the water phase to advance more easily. Consequently, the concave front edge gradually transforms into a jerky-shaped convex front edge. This change in shape indicates that the water phase is now able to displace the gas phase more efficiently, resulting in a smoother and more continuous displacement process. These observations highlight the dynamic nature of the replacement process and the influence of the gas phase’s volume fraction on the displacement resistance. The initial concave shape and subsequent transformation to a convex shape provide insights into the evolving flow patterns and the interplay between the gas and water phases during the seepage process in undisturbed soil.

**Figure 14:**
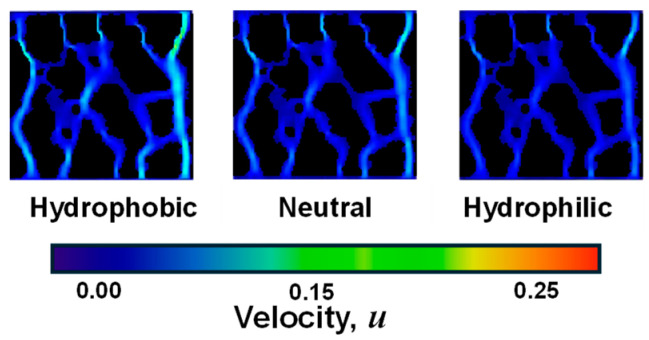
Water-phase seepage velocity field of GRS models with different wall wettability during seepage stabilization.

When examining the two-phase seepage process under various wall-wetting conditions (hydrophobic, neutral, and hydrophilic), it was discovered that the “finger-in” phenomenon occurred during the replacement process across all conditions in Figure 14. Additionally, the flow velocity varied significantly between different pores, with a more pronounced preferential flow effect in straight and larger pores. In a porous media model, an increase in wall hydrophobicity led to an increase in two-phase seepage velocity. This means that the stronger the hydrophobicity of the soil pore wall, as indicated by a larger wetting angle, the faster the corresponding flow rate [47]. The angle between the gas–liquid and solid–liquid interface lines at the intersection of the solid, water, and gas phases on the pore wall surface is commonly used to determine the hydrophilic or hydrophobic nature of the pore wall surface, as well as its strength. A 90° wetting angle is considered the critical point for the pore wall surface to exhibit both hydrophilic and hydrophobic properties. For hydrophobic walls, the seepage velocity will be accelerated to varying degrees depending on the strength of the hydrophobicity of the wall. This is because the wall will have a repulsive effect on the neighboring fluids. When the wall is hydrophobic, it repels water molecules, leading to a reduced interaction between the fluid and the wall surface. This repulsive effect reduces the resistance to fluid flow and allows for a higher seepage velocity in the presence of hydrophobic walls. Conversely, for hydrophilic walls, the seepage velocity will be decelerated to a certain extent due to the viscous effect produced by the hydrophilicity of the wall. Hydrophilic surfaces have a stronger affinity for water molecules, which leads to increased adhesion and a higher resistance to fluid flow. This viscous effect results in a decrease in seepage velocity when fluids come into contact with hydrophilic walls.

The influence of wall hydrophobicity or hydrophilicity on seepage velocity has been studied in various contexts. For example, in microfluidic systems, the surface properties of channels and walls play a crucial role in determining fluid flow behavior [6,14,49,50]. Studies have shown that hydrophobic channels promote faster flow rates, while hydrophilic channels hinder fluid flow due to increased viscous resistance. Understanding the effects of wall hydrophobicity or hydrophilicity on seepage velocity is essential for various applications, such as filtration processes, fluid transport in porous media, and the design of microfluidic devices. By controlling the surface properties of walls, engineers can manipulate the seepage velocity and optimize fluid flow for specific purposes.

**Figure 15:**
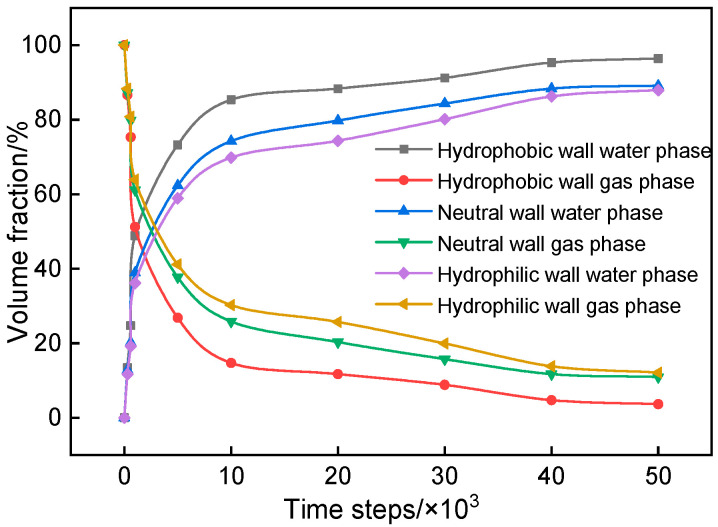
Change of the two-phase volume fractions of the models with different wall wettability at different time steps (unit: step).

Figure 15 elucidates the relationship between the water/gas volume fractions and flow time within the model. The analysis reveals that with the progression of two-phase percolation time, there is a marked increase in the water-phase volume fraction, while the gas-phase volume fraction demonstrates a decline before stabilizing. Notably, the apex of the water-phase volume fraction is observed under hydrophobic wall conditions, reaching up to 96.40%, with the residual gas-phase volume fraction constituting 3.60%.

This phenomenon underscores the impact of wall wettability on the volume fractions, with the sequence of residual gas-phase volume fraction under varying wall conditions being hydrophilic wall > neutral wall > hydrophobic wall. As mentioned previously, the wettability of the wall surface influences the adhesion and interaction between the fluid phases and the wall, leading to variations in the distribution of gas and water within the porous medium. Hydrophilic walls have a stronger affinity for water, resulting in a higher residual gas-phase volume fraction compared to hydrophobic walls. Conversely, the rate at which the gas phase is supplanted by the aqueous phase follows a different sequence: hydrophobic wall > neutral wall > hydrophilic wall. This indicates that the wettability of the wall also affects the displacement dynamics of the fluid phases. Hydrophobic walls facilitate the displacement of the gas phase by the aqueous phase more rapidly compared to hydrophilic walls. Furthermore, it was ascertained that the cumulative volume fractions of the water and gas phases within the model remained invariant over time, aligning with the principle of mass conservation, with both fractions jointly totaling one [49].

### 5.4. Summary

The results of the study demonstrate the influence of pore structure, porosity, and wall wettability on the two-phase seepage behavior within pore structures of U-RGS.

In terms of pore structure, the water-phase seepage initially follows a “finger-in” progression, preferentially infiltrating larger, more direct channels before resorting to narrower, more tortuous pathways. This phenomenon can be attributed to the narrowness of certain pores, which impedes the ingress of water, as well as the presence of adjacent wider and straighter channels that water preferentially migrates through. This preferential flow effect has been observed in previous studies [9,47], where water flows through larger and more connected channels while gas is displaced and trapped in smaller pores. The presence of water in the channels also obstructs the expulsion of the gas phase, causing it to be trapped in smaller pores. This finding is consistent with sporadic appearances of free water fluids within the gas-phase domain during the two-phase flow process [49]. The gas–water phase flow persists within the pores until it either merges with the main water body or exits the soil matrix, exerting a relatively minimal displacement impact on the gas phase. This behavior indicates that the displacement of the gas phase by the water phase is not uniform and can be influenced by the pore structure and the preferential flow paths. Therefore, aforementioned findings highlight the complex dynamics of two-phase flow in undisturbed soil and the need to consider the interplay between pore geometry and fluid behaviors for a comprehensive understanding of the displacement process.

Regarding porosity, the overall velocity of the water phase decreases as the porosity of the model decreases. However, the lack of a significant correlation between preferential flow and porosity highlights the importance of considering the spatial distribution and connectivity of pores when studying two-phase flow in undisturbed soil. It indicates that the local seepage velocity is closely related to the pore structure morphology, and the generation of the preferential flow is not significantly correlated to the porosity [9,47,48]. Pore structure characteristics, such as the presence of narrow or wide channels, can have a significant impact on the flow behaviors and the preferential migration of fluids. Therefore, a comprehensive characterization of the pore structure morphology is crucial for understanding and predicting the flow dynamics in undisturbed soil. These findings have important implications for soil management and engineering applications. Understanding the preferential flow behavior and its relationship with pore structure can help in designing more efficient drainage systems, optimizing irrigation practices, and predicting contaminant transport in soil. By considering the specific pore structure characteristics, engineers and researchers can develop more accurate models and simulations to predict and mitigate potential flow-related issues in soil systems.

Finally, the influence of wall wettability on volume fractions is also evident. The residual gas-phase volume fraction follows the sequence of hydrophilic wall > neutral wall > hydrophobic wall, indicating the impact of wall wettability on fluid distribution within the porous medium. The rate at which the gas phase is supplanted by the aqueous phase follows a different sequence: hydrophobic wall > neutral wall > hydrophilic wall. This highlights the importance of the interaction between fluid phases and the wall surface in determining the displacement dynamics. The influence of wall hydrophobicity or hydrophilicity on seepage velocity has been studied in various contexts. For example, in microfluidic systems, the surface properties of channels and walls play a crucial role in determining fluid flow behavior [6,14,49,50]. Studies have shown that hydrophobic channels promote faster flow rates, while hydrophilic channels hinder fluid flow due to increased viscous resistance. Understanding the effects of wall hydrophobicity or hydrophilicity on seepage velocity is essential for various applications, such as filtration processes, fluid transport in porous media, and the design of microfluidic devices. By controlling the surface properties of walls, engineers can manipulate the seepage velocity and optimize fluid flow for specific purposes.

## 6. Conclusions

This study utilized X-ray CT and the lattice Boltzmann method (LBM) with the Shan–Chen model to simulate the impact of internal pore characteristics of undisturbed granite residual soil on water–gas two-phase seepage flow behaviors at the microscale. The investigation examined the effects of pore structure, porosity, and wall wettability on the replacement process. The major conclusions can be found as follows:

The leading edge of two-phase seepage is “finger-in” forward, and the fluid prefers larger, straight pores for seepage before choosing narrower, curved pores. Additionally, there is a residual gas phase in the pore space, mainly distributed in the pore edges and dead ends.

The maximum value of two-phase seepage velocity is positively correlated with porosity, but the local seepage velocity is closely related to the distribution and complexity of the pore structure. This highlights the importance of considering the spatial distribution and connectivity of pores when studying two-phase flow in undisturbed soil. Therefore, a comprehensive characterization of the pore structure morphology is crucial for understanding and predicting the flow dynamics in undisturbed soil and also for implications for soil management and engineering applications, such as drainage system design, optimizing irrigation practices, and predicting contaminant transport in soil.

The wettability of the pore wall has a significant influence on the two-phase seepage process, with the seepage velocity increasing with hydrophobicity. The water-phase volume fraction shows a sharp increase in the initial stage of two-phase seepage, with the maximum value occurring under the hydrophobic wall condition.

Overall, this novel study suggests an effective nondestructive approach, provides insights into the dynamic visualization of immiscible two-phase seepage in soil, and contributes to the development of effective strategies for soil management and engineering applications. Based on the findings, it is recommended for engineers in the industry to consider the spatial distribution and connectivity of pores when designing and implementing two-phase flow systems. The presence of preferential flow paths and the impact of wall wettability should be taken into account to ensure efficient fluid displacement and management. Finally, future studies can consider further investigating the complex interactions between pore structure, porosity, and wall wettability. Advanced imaging techniques and modeling approaches can be employed to gain a deeper understanding of the underlying mechanisms and to develop more accurate predictive models. Additionally, exploring the impact of other factors, such as temperature, pressure, and chemical interactions, on two-phase seepage behavior would enhance our knowledge and enable the development of more robust engineering solutions.

## Figures and Tables

**Table 1:** Validation example calculation parameters (lattice unit).

*A*	*ρ*_1_	*ρ*_2_	*G*_1_	*G*_2_	*G*_3_	*δ_x_*	*δ_t_*
200	0.6	0.4	1.2	1.4	1.6	1.0	1.0

Notes: *A* is the side length of the computational model; *ρ*_1_ and *ρ*_2_ are the initial densities of the two phases, respectively; *G_i_* is the strength of the force between the two components in the three cases, respectively; *δ_x_* and *δ_t_* are the grid step and time step, respectively.

**Table 2:** Two-phase flow calculation parameter table (Lattice unit).

*ρ*_1_	*ρ*_2_	*G*_w_	*G*_wr_	*G*_wb_	*δ_x_*	*δ_t_*	*u*
0.001	1.0	1.4	0.2	−0.2	1.0	1.0	0.2

## Data Availability

Data are contained within the article.
